# Comet Pond II: Synergistic Intersection of Concentrated Extraterrestrial Materials and Planetary Environments to Form Procreative Darwinian Ponds

**DOI:** 10.3390/life8020012

**Published:** 2018-05-11

**Authors:** Benton C. Clark, Vera M. Kolb

**Affiliations:** 1 Research Branch, Space Science Institute, Boulder, CO 80201, USA; 2 Department of Chemistry, University of Wisconsin–Parkside, Kenosha, WI 53141, USA; kolb@uwp.edu

**Keywords:** origin of life, comets, comet pond, prebiotic synthesis, extraterrestrial organics, freeze/thaw, wet/dry, Darwinian pond

## Abstract

In the “comet pond” model, a rare combination of circumstances enables the entry and landing of pristine organic material onto a planetary surface with the creation of a pond by a soft impact and melting of entrained ices. Formation of the constituents of the comet in the cold interstellar medium and our circumstellar disk results in multiple constituents at disequilibrium which undergo rapid chemical reactions in the warmer, liquid environment. The planetary surface also provides minerals and atmospheric gases which chemically interact with the pond’s organic- and trace-element-rich constituents. Pond physical morphology and the heterogeneities imposed by gravitational forces (bottom sludge; surface scum) and weather result in a highly heterogeneous variety of macro- and microenvironments. Wet/dry, freeze/thaw, and natural chromatography processes further promote certain reaction sequences. Evaporation concentrates organics less volatile than water. Freezing concentrates all soluble organics into a residual liquid phase, including CH_3_OH, HCN, etc. The pond’s evolutionary processes culminate in the creation of a Macrobiont with the metabolically equivalent capabilities of energy transduction and replication of RNA (or its progenitor informational macromolecule), from which smaller organisms can emerge. Planet-wide dispersal of microorganisms is achieved through wind transport, groundwater, and/or spillover from the pond into surface hydrologic networks.

## 1. Introduction and Background

**Figure 1:**
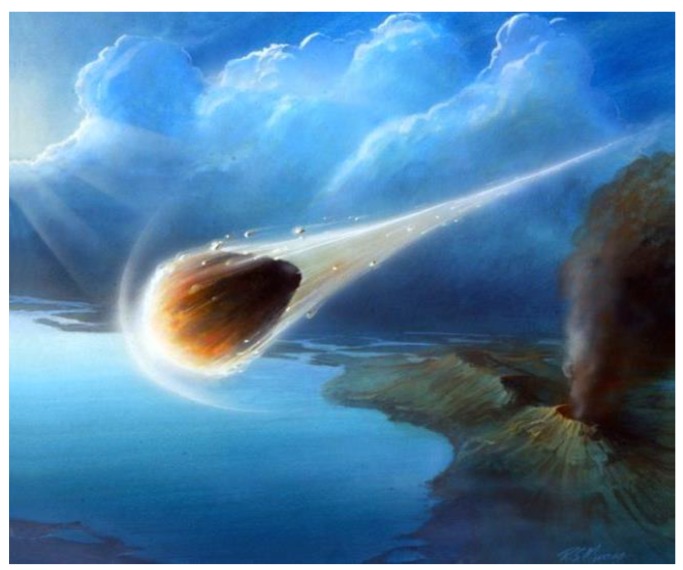
A cometary nucleus or primitive carbonaceous planetoid accomplishes a rare entry event which allows the survival of pristine organic material and ices upon landing on the surface of a planetary body, such as Earth. (Credit: Lockheed Martin).

**Figure 2:**
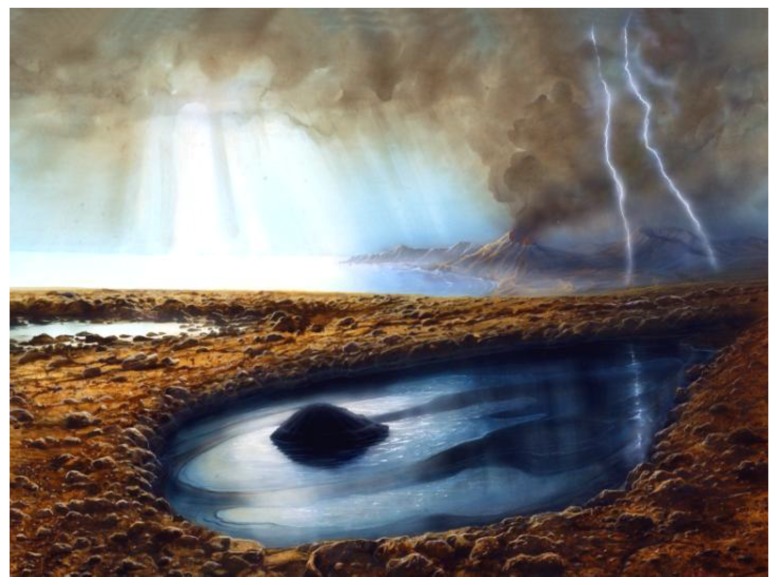
Following a relatively low-velocity landing, the ices melt to form a pond with a residual organic-rich central mound as well as bottom sludge, plus liquid with solutes and colloidal particulates, and surface scum. The pond interacts with surface minerals and atmospheric gases to create ideal conditions for diverse prebiotic syntheses. (credit: Lockheed Martin).

The “comet pond” conjecture [1] envisions the landing of a relatively pristine portion of a cometary nucleus that could have provided ideal starting materials and conditions for prebiotic syntheses leading to the origin of life on Earth. A comet transits the earth’s atmosphere, Figure 1, perhaps at a highly oblique angle, which maximizes the path length before reaching the surface, and is slowed sufficiently for a relatively soft impact into weak regolith. This forms a depression which, upon melting of its remaining constituent ices, creates a pond of highly concentrated organic-rich matter, Figure 2.

Modern understanding of the very early evolution of the solar system and the state of the atmosphere of the early Earth, as well as new information on the uniquely diverse composition of comets, can now be applied to this hypothesis. Furthermore, the concept can be broadened to take into account more fully the interaction of the comet pond with geochemical ingredients and physicochemical properties indigenous to the target planet. Finally, scenarios for the spread and colonization of the planet beyond the original pond will be examined.

The early speculation by Darwin [2] that only a single little pond might have been the locale for the origin of life was formulated long before there was any scientific basis for the occurrence of small bodies of water that could have been organic-rich. Pathways for the creation of complex organic molecules on the early Earth, derived from inorganic gaseous precursors due to the action of electric discharges simulating lightning [3], provided the rationale for the advent of organic material. Coupling this with volcanic hydrothermal activity [4] to produce a greater range of amino acids and other molecules of prebiotic relevance provided a further basis for the concentration of reactants that might fuel “a warm little pond”. 

The discoveries that extraterrestrial materials such as carbonaceous meteorites, comets, and grains in the interstellar medium are themselves endowed with complex organic matter, including amino acids, provided an alternative scenario for the development of organic-rich ponds. We proposed the “comet pond” model as a way to create such a pond [1], even though it would be a very low probability event due to the challenges of landing pristine cometary material. With the low tensile strength of cometary nuclei, they generally experience rapid destruction and dispersal from entry through the atmosphere or hypervelocity impact onto the planet’s surface. Separately, detailed analyses indicate that cometary material could have provided some amount of organics to the early Earth due to the widespread bombardment in the early history of the Earth’s surface [5].

Pond scenarios for the origin of life have recently been receiving greater attention [6,7,8,9,10]. New discoveries of comet composition provide further support for the potential importance of pristine cometary material being involved in the origin of life on Earth, as discussed in Section 2 below.

As originally proposed, the event whereby cometary matter is preserved in reaching the surface of the Earth is very rare compared to the fate of comets which impact Earth in general. Several mechanisms by which this might happen are proposed [1].

For the small cometary nucleus, less than 0.1 to 1 km [11], the object will explode once the pressure differential across the body is stronger than its inherent tensile strength, which is typically taken as a low value of less than 0.1 MPa. Data from the recent Rosetta mission to comet 67P/Churyumov-Gerasimenko (hereafter, 67P/C-G) have been interpreted to indicate a near-surface hard layer with a compressive strength of 4 MPa [12], based upon the results of the Philae lander.

Larger objects do not sufficiently slow in the atmosphere for this to occur [5], and therefore impact the surface at hypervelocity, which destroys much of the material and disperses it over a large region. As seen from meteorite strewn fields, impact crater ejecta patterns, and the occurrence of impact melt, there is little chance that significant amounts of cometary organics could survive and populate one specific area. Nonetheless, some organic molecules are expected to survive in their original form [5]. Over time, with large numbers of cometary impacts, a significant inventory of organic material may be delivered. However, because comets did strike the planetary surface in a stochastic manner, there is a vanishingly low probability of two or more comets impacting the same area in a geologically short time interval. During this intervening time, the critical organic material from an earlier strike may have become dispersed, destroyed, or buried by weathering and/or diagenetic processes.

### 1.1. The Dilution Issue

From planetary formation scenarios and isotopic signatures, comets are not considered sufficient to provide all the water to account for the Earth’s ocean [13]. Thus, any cometary delivery of water and organics will result in an organic concentration below that in the initial comet pond. Because the ocean is vast, any one comet will have an extremely small or entirely negligible effect. Likewise, if the cometary material falls on land, the effects of rainfall and network draining will further disperse the organic components. These spatial dilution effects are a primary objection to the concept of a steady build-up of organics as the progenitor condition for the origin of life.

This brings up that, in addition to the spatial dilutive effect, there is another factor of dilution caused by the time intervals between cometary deliveries. When organics are delivered, they will not only be affected by entry pyrolysis, but by subsequent alteration, as they reside on the surface of the planet they strike. They will be affected by the leaching process of water, extracting and diluting soluble components. Rainwater and wind will disperse material on short timescales compared to the next comet impact. Susceptible organics will also be subjected to hydrolysis reactions that can destroy larger molecules. Other weathering processes can separate organics from unique minerals also brought in by the comet, and alter such minerals to less useful forms, converting much of the chemistry from highly activated, non-equilibrium states to ones which become non-reactive. Because the early Earth was devoid of significant O_2_ in the atmosphere, there was no ozone shield and exposed organics could suffer from cross-linking and other degradative processes by extreme ultraviolet (EUV) photons from the Sun and ionizing radiation from cosmic rays.

We term this “double-dilution”, whereby the organics delivered are less useful because of both spatial and temporal dilutive effects. Because the impacting bodies cannot be physically large compared to the planetary object without temporarily creating inhabitable conditions (e.g., evaporating the oceans and melting surface rocks), the spatial dilution effect is large. For example, the contribution of a bolide of radius r of material averaged over a whole planet of radius R can be evaluated by the simple relationship,

(r/3t) × (r/R)^2^ (1)

where t is the thickness of the ocean or of the regolith gardened by micrometeorites.

Even for an extremely large nucleus, such as one that is 100 km in diameter, the cometary matter would contribute only 0.03% to the mass of the ocean. However, at that size (~10× the Chicxulub impactor’s estimated diameter) and speed of impact, organic covalent bonds could have been broken for most of the molecules. For impactors of size 10 km or so, the contribution to the top one meter of regolith could have averaged 0.1% globally. This, too, is small compared to what may be needed for a vigorous prebiotic chemistry.

In a similar calculation, if a 100 m cometary nucleus or fragment thereof is subject to disintegration or an explosive airburst at altitude (~20–30 km), its matter will disperse. If its material spreads over a typical strewn field area of, say, 50 sq. km, the contribution to the regolith or water to a depth of 1 m would be only 0.1% by weight. Objects larger than ~1 km diameter will not be sufficiently slowed to avoid the hypervelocity impact on the surface, resulting in pyrolytic destruction of compounds and/or widespread dispersal [5,11]. This is another fruitful area for future additional analyses and modeling.

In a given location, multiple impacts from different primary bodies are rare. For a planet, the probability of a random impact within 1 km within any given spot is the ratio of the area of that location to the surface area of the planet. For Earth, this is just slightly over one chance in one billion, (1.5 × 10^−9^). Thus, the probability of another impactor being this close to the first one is less than 1 × 10^−17^. With this spread over a time period of one-half billion years, the probability of an impact that is close in both time *and* location is vanishingly small, even with a large number of impactors. The accumulation of exogenous material in one location is therefore not to be expected.

This calculation does not reflect the fact that because of breakup during entry or production of secondaries, there can be crater clusters in the geologic record. However, these patterns of close impacts are due to a single original bolide, and do not reflect the cumulative effect of the overall bombardment. The spread in both space and time for any given location on the planet to receive more than one comet or asteroid impact is vast, and the basis of the double-dilution effect.

### 1.2. Comets and Cometoids

Finally, we should observe that the typical comets are bodies in generally eccentric orbits with aphelia far from the Sun and which originally were in the zone of the outer planets where temperatures are extremely cold. However, with the discovery of the unanticipated composition of the asteroid Ceres [14] and that certain bodies in the classical asteroid Main Belt zone have been observed to actively emit gas and dust [15], attention has been focused on objects not just in the typical cometary regions. These Main Belt comets, and other “cometoids”, including various trans-Neptunian objects, are also candidates for the comet pond scenario, if their organic components surpass those of carbonaceous chondrites and rival or equal those of the more classical cometary bodies. 

## 2. New Results and Developments

### 2.1. Solar System Evolution

A recent revolution in the concepts of the dynamic instability of the early solar system and re-configuration due to migration of the giant planets has come from insights gained through detailed, higher-fidelity calculations of the gravitational interactions of planetary and smaller bodies. The Grand Tack model [16] describes early inward migration of Jupiter followed by subsequent outward migration, and the Nice Model [17] includes clearing of much of the contents of the trans-Neptunian region of its initial very large population of ice-rich objects. Of particular relevance here are the envisioned scenarios at the time of the Late Heavy Bombardment (LHB)—the period in which there appears to have been an unusually large increase in impact rates on the inner planets, as revealed by craters on the surfaces of the Moon and Mercury

Based on extensive simulations, in a very recent analysis, Morbidelli et al. [18] conclude that during the LHB, the impacts by comets on the Earth and Moon would actually exceed impacts by asteroids, a circumstance much reversed in later epochs. Efforts are underway by laboratory scientists seeking evidence of this increased injection of more primitive objects into the inner solar system [19].

However, Morbidelli et al. [18] also discuss why the LHB may actually be more perceptual than real. If the cratering rate was actually monotonically decreasing during the period around 4 Gy assigned to the LHB, to produce an “accretion tail”, those impactors must have been predominantly asteroidal. A deficiency in platinum-group elements in lunar surface material does seem to indicate a much lower contribution by comets than asteroids [20]. In either case, however, the impacts by comets are not negligible and only a single impactor which fulfills the conditions of entry survival and soft landing needs to occur in order to realize a comet pond hypothesis.

### 2.2. Comets: Chemical Composition and Physical Properties

Comets are typically considered to consist of small grains of organic and silicate matter, held together by ices formed from more volatile material (H_2_O, CO_2_, etc.). It is well known that Interplanetary Dust Particles (IDP’s) and especially interstellar (IS) particulates are extraordinarily fine-grained, less than 0.1 μm, compared to minimum particle sizes in typical planetary regoliths. These particles can extend down to nanometer size scales [21]. Dust particles of a sub-micron size from comet 67P/C-G have also been observed, although there are also large aggregates of a high porosity and low strength, but made up of smaller particulates [22].

To the extent that interstellar particles and grains formed in the primitive protosolar disk are also small, these tiny grains can preserve their native size if isolated from one-another by ices. As comets traverse closer to the Sun, they release large aggregates of ice and particulates, which then further disintegrate and liberate the non-volatile grains that show a steep size distribution favoring smaller sub-micron particles [23,24].

Such particles have high chemical reactivity or catalytic functionality, and could initially create a dense colloidal solution in the comet pond. Once particles are reduced to the 1–10 nm size range, their physical and chemical properties can change dramatically, exhibiting neither those of the bulk material nor those of their individual molecules, as described in the literature [25].

Several missions to comets (Stardust, Deep Impact, EPOXI, Stardust NExT, and Rosetta) have made additional important new discoveries relevant to the comet pond hypothesis. For example, the Stardust sample return mission from comet Wild 2 made the surprising discovery that although comets contain abundant low-temperature matter, they also contain a variety of minerals which can form only at high temperatures much closer to the Sun than the current location of cometary bodies [26].

From past and current studies, much has been learned or can be presumed about the physical and chemical make-up of the cometary nucleus. First among these in terms of importance are the organic constituents, which may serve as the starting “broth” for the primordial “soup”. To the extent that key inorganic constituents, such as minerals and the elements within them, are already in abundant supply from terrestrial soil, they may make important contributions to the comet pond milieu. Finally, we consider ways in which the physical nature of the non-volatile grains and the icy matrix may contribute to the unique characteristics of the comet pond.

#### 2.2.1. Organics

In this section, we address organic molecules, especially those which are significant as precursors for prebiotic synthesis which led to life, and which were brought to the early Earth from space. Although our focus is on the organics from comets, we must also address organics from space in general, since data for organics in comets are still very limited. This deficiency will hopefully be remedied by future comet sample return missions.

Numerous organic molecules are present in space. At this time, over 200 molecules, the majority of them organic, have been detected in the gas phase in the interstellar medium or circumstellar shells (https://www.astro.uni-koeln.de/cdms/molecules). Detected species include diverse groups of organic molecules, such as hydrocarbons (alkanes, alkenes, and alkynes), alcohols, aldehydes, ketones, carboxylic acids, amines, amides, and ethers, among others ([27,28]). Kwok also discusses the possibility that the early Solar System and eventually the early Earth have been enriched by these interstellar organics. This may pertain mainly to organic phases since the results of analysis of the particles successfully analyzed by the Stardust mission (mainly non-organic minerals >2 microns) indicate relatively little contribution of minerals in comet Wild 2 from interstellar grains [26].

Meteorites of carbonaceous chondrite types are reservoirs of a variety of organics. As one example, over 14,000 organic species, comprising a much larger number of diverse structures, have been identified in the Murchison meteorite [29,30,31]. Chemical groups include aliphatic and aromatic hydrocarbons, alcohols, aldehydes, ketones, carboxylic acids, sugar-related compounds, sulfonic and phosphonic acids, amino acids, and heterocyclic compounds.

Comets contain organic materials either as frozen volatile molecules or as solid-state refractory substances. A large number of organics have been detected in the gas phase, containing C, H, O, and N atoms. Citing previous work, many representative interstellar organic and inorganic molecules are potentially relevant to prebiotic synthesis and the origin of life. They include: methane (CH_4_), ethane (C_2_H_6_), methanol (CH_3_OH), hydrogen sulfide (H_2_S), ethylene glycol (HO-CH_2_CH_2_-OH), formaldehyde (H_2_CO), acetylene (H_2_C_2_), acetaldehyde (CH_3_CHO), formic acid (HCOOH), methyl formate (HCOOCH_3_), formamide (H_2_NCHO), hydrogen cyanide (HCN), isocyanate (HNCO), acetonitrile (CH_3_CN), and ammonia (NH_3_) [32].

The amino acid glycine was found [33] in craters in the metal foils on the Stardust collector array. Unfortunately, the detection and analysis of organics in the Stardust aerogel collector is restricted because although considerable efforts were made to reduce the organic background, these efforts were of limited success because the manufacture of the silica foam aerogel began with an organic process. Furthermore, because of the high impact velocity (6.1 km/s), much of the organic material in particles was apparently rapidly volatilized, dispersed, and redistributed deeply into the aerogel at distances far from the track of the particle that contained it [34]. However, it was nonetheless possible to determine that the organic-rich particles are generally different from most grains in carbonaceous chondrites, containing material more labile to impact heating, more diverse in their constituents, and that contain higher levels of heteroatom molecules (higher N/C and O/C ratio’s) than meteorite organics [34,35]. Real-time analysis of particle compositions during passage through the coma also revealed the presence of N-rich organics [36].

At comet 67P/C-G, the following molecules were detected in the gas phase [37]: methane (CH_4_), ethane (C_2_H_6_), propane (C_3_H_8_), butane (C_4_H_10_), ammonia (NH_3_), water (H_2_O), hydrogen cyanide (HCN), carbon monoxide (CO), carbon dioxide (CO_2_), methanol (CH_3_OH), ethanol (CH_3_CH_2_OH), propanol (C_3_H_7_OH), ethylene glycol (CH_2_(OH)CH_2_(OH)), formaldehyde (H_2_CO), acetaldehyde (C_2_H_4_O), acetone (C_3_H_6_O), acetic acid (CH_3_COOH), methylamine (CH_3_ NH_2_), propylamine (C_3_H_7_NH_2_), isocyanic acid (HNCO), formamide (HCONH_2_), acetamide (CH_3_CONH_2_), thioformaldehyde (CH_2_S), formic acid (HCOOH), methanethiol (CH_s_SH), and ethanethiol/dimethylsulfide (C_2_H_6_S). These volatile organics are only a rough indicator of the more complex organics which are presumably present in cometary bodies and contain important atoms such as N, O, and S.

Methyl chloride (CH_3_Cl) was recently reported for comet 67P/C-G [38]. Glycine has also been detected, in the gas phase [39], complementing the discovery of comet Wild 2 by Stardust.

The composition of particles collected by the Rosetta spacecraft inside the COSIMA instrument [40] indicates that the dust from comet 67P/C-G is nearly 50% organic matter by mass, which emphasizes the uniqueness of comet chemistry. The C/Si ratios (atom/atom) range from 3:1 to 8:1 in a population of 30 large particles analyzed with a high accuracy that can be compared to <1:1 for the same ratio in carbonaceous chondrite meteorites. These results were reported only for the larger particles (50–1000 microns in diameter) of the 250 particles analyzed during the mission because the 40 micron ion beam could have its field-of-view filled, thereby avoiding measurement of contamination on the supporting plate. Smaller particles may have an even higher organic content, analogous to the small, highly organic-rich CHON particles detected at comet Halley [41].

Antarctic micrometeorites are found to be very organic rich, a small population of which contains up to 90% organics [42], designated as UCAMMS (Ultracarbonaceous Antarctic Micrometeorites). Minerals associated with these particles include olivine, Fe sulfides, and GEMS (see below). The fragile UCAMMS are dominated by N-rich organic matter, with their high organic content signaling a long residency time in the outer portion of the solar system [43] and hence a possible association with cometary bodies.

As has briefly been reviewed here, complex organics from space and likely to be in comets contain major chemical functional groups, and thus exhibit great prebiotic chemical potential.

#### 2.2.2. Inorganics

From the Stardust sample return mission, the overall elemental composition of the inorganic component of cometary particulates has been shown to mimic the composition of the Sun and carbonaceous chondrites [44]. Thus, there is an ample supply of many important trace elements with important catalytic activity that could become available in the comet pond “soup”, as detailed previously [1]. Solubilizing minerals to release catalytic ions is favored by the generally reduced conditions in Earth’s primitive atmosphere and by the extremely small grain size of many cometary constituents.

Wild 2 is a remarkable mixture of different minerals, with some being exotic compared to terrestrial or meteoritic occurrences. Olivine grains are abundant, and together with pyroxenes, are analogous to the fundamental composition of igneous mafic rocks. Chondrules, which have been highly processed at high temperature, are also present. Various materials which may have special importance include iron sulfides, phosphorus minerals, and minerals composed of trace elements.

##### Fe and Fe-S

In enzymatic biochemistry, the Fe-S ligand plays a variety of important roles. The hydrogenases catalyze the reversible oxidation of molecular H_2_, a key metabolite potentially available in the atmosphere of the early Earth. In contemporary biology, hydrogenases are widespread across the microbial world. Although there is one type of hydrogenase whose active site only contains Fe, the more common ones contain multiple Fe-S clusters, sometimes also including Ni. Other important iron-sulfur proteins include the ferredoxins, certain dehydrogenases, nitrogenase, reductases, and coenzyme Q. All contain structural motifs of Fe-S clusters in various multiplicities.

It is reasonable to draw the analogy to primitive early catalysis of these functions if there are Fe-S minerals which may be available in the comet pond. This is eminently the case because the comet provides minerals such as pyrrhotite (FeS_x_) and pentlandite (Fe,Ni)_9_S_8_ [26]. Even native metal such as FeNi_x_ occurs in comets, as well as being common in many asteroidal meteorites. One major type of IDP, designated the chondritic porous (CP) class, contains the unusual submicron aggregates called GEMS, for Glass with Embedded Metals and Sulfides. Many Stardust grains from comet Wild 2 also seem to include this material. The GEMS particles are themselves made up of nanometer-sized sub-grains of kamacite (FeNi metal) and Fe-Ni sulfide in a Mg-Fe-Al amorphous silicate matrix. They may be unique to comets compared to asteroids, as they are not found in meteorites. Their tiny size, porous structure, and fragile nature could allow them to react rapidly and thoroughly in an aqueous milieu. Furthermore, because of their high porosity, GEMS are under-dense compared to their crystalline equivalent [45] and hence might float in water.

##### Phosphorus

The element P is well recognized to play key roles across all of the most important characteristics of terrestrial living systems: genetics (DNA and RNA): energy metabolism (ATP); compartmentalization (phospholipids); and biosynthesis of proteins (RNA’s). Furthermore, it is now recognized that these and other roles must be deep seated in the history of life [46]. The geochemical availability of phosphorus has been worrisome because the most stable P minerals at the Earth’s surface are apatites, which resist alteration by water and release of usable P.

It was proposed in 2005 that the extraterrestrial mineral schreibersite may have been the original source of available P atoms [47] because the Fe-Ni phosphide is readily altered into phosphites and phosphates. Other schemes for obtaining P atoms have been considered in detail but this remains the most highly promising source [48]. Schreibersite is one of the minerals which occurs in comet Wild 2, as shown directly by the Stardust sample return mission [26]. In addition, two minerals which include Ca phosphate, whitlockite, and merrillite, have also been found in the Stardust particle collection. For comet 67P/C-G, phosphorus has been detected in the coma by the ROSINA mass spectrometer, although the volatile parent molecule has not been determined [39].

Interestingly, it has also been suggested that if phosphorus were not sufficiently accessible, there could be a core metabolism scheme utilizing enzymes with Fe-S clusters [49]. Thus, the comet pond has an alternative or supplemental opportunity for mimicking many of the key functions of phosphate in the earliest phases of prebiotic chemical evolution.

##### Trace Minerals and Elements

As pointed out, the comet is a complex assortment of not only organic compounds, but also minerals of various types. Although some are rare, they may enable critical functions. It should be borne in mind that although the pond is not large, and unusual minerals are a small component, on the size scale of microbes, many of these are comparable in size (micron or sub-micron), and on the scale of molecular reactions, they are enormous.

Many grains found in comet Wild 2 are of minerals which are rare on Earth [26] but might be of prebiotic significance because of the unusual elements they contain. For example, cubanite (CuFe_2_S_3_), could be a source of Cu-S ligands, which participate in biological electron transfer reactions [50] and the Cu catalyst for the Sutherland scheme syntheses [9]. In addition to copper, zinc is also enriched in Wild 2 particles, compared to carbonaceous chondrites [44]. Zinc has also been reported as the sulfide, which could be important because Zn is one of the most used transition elements in enzymatic reaction centers, after Fe.

Osbornite (TiN) grains sometimes have a high vanadium content. The biological uses of V are multiple and significant [51], although the osbornite examples of the Stardust mission are occluded inside anorthite and spinel.

The kosmochloric pyroxene grains contain high concentrations of Cr. Micro-nuggets of Pt-group elements are also found. Low-iron but manganese-enriched (LIME) olivines could supply binuclear Mn-based enzymatic activity, which can catalyze a variety of reactions.

These examples are only the result, so far, of compositional analysis of approximately ten percent of the thousands of grains that the Stardust mission returned to Earth in its aerogel collectors. Additional exotic but potentially significant mineral types may be discovered in future analyses. 

### 2.3. Early Earth Environment

Because of the faint early Sun [52], it has been presumed that the Earth was originally much colder than it is today, with solar insolation being lower by one-third or so. With the positive feedback toward less absorption of solar flux due to the high albedo of snow and ice covering rock and soil, the Earth could have become a “snowball” planet until the climate warmed. The geological evidence against glaciation in the earliest epoch has invoked atmospheric models which incorporate strong greenhouse warming to prevent the transition to the snowball state. However, Charnay et al. [53] predict that cold climates could occur after large impacts during the heavy bombardment. 

The early atmospheric models also assume much higher pressures than today’s 1-bar because of the availability of large quantities of CO_2_ that have since been sequestered into widespread carbonate deposits. From new evaluations of the enhanced greenhouse in a nitrogen-rich atmosphere with H_2_, the H_2_-N_2_ collision-induced absorption allows a warmer Earth with smaller amounts of CO_2_ in the atmosphere [54,55,56]. Thus, it is unclear whether the Earth atmosphere at the time of the origin of life may have been in the range of 10, 20, or more bar [57], or closer to today’s pressure of somewhat less than one bar of N_2_. 

The chemical composition of the early atmosphere is also questionable, especially its redox state, which is probably not strongly reducing [58]. Volcanism under various conditions produces a range of gases which include H_2_S, SO_2_, HCl, CO, CO_2_, CH_4_, etc. However, Ranjan et al. [59] found that multiple factors limit the availability of H_2_S in shallow lakes or ponds. 

### 2.4. Comet Pond Description

In this paper, we present an updated and expanded model for the comet pond.

#### 2.4.1. Comet Pond Description

**Figure 3:**
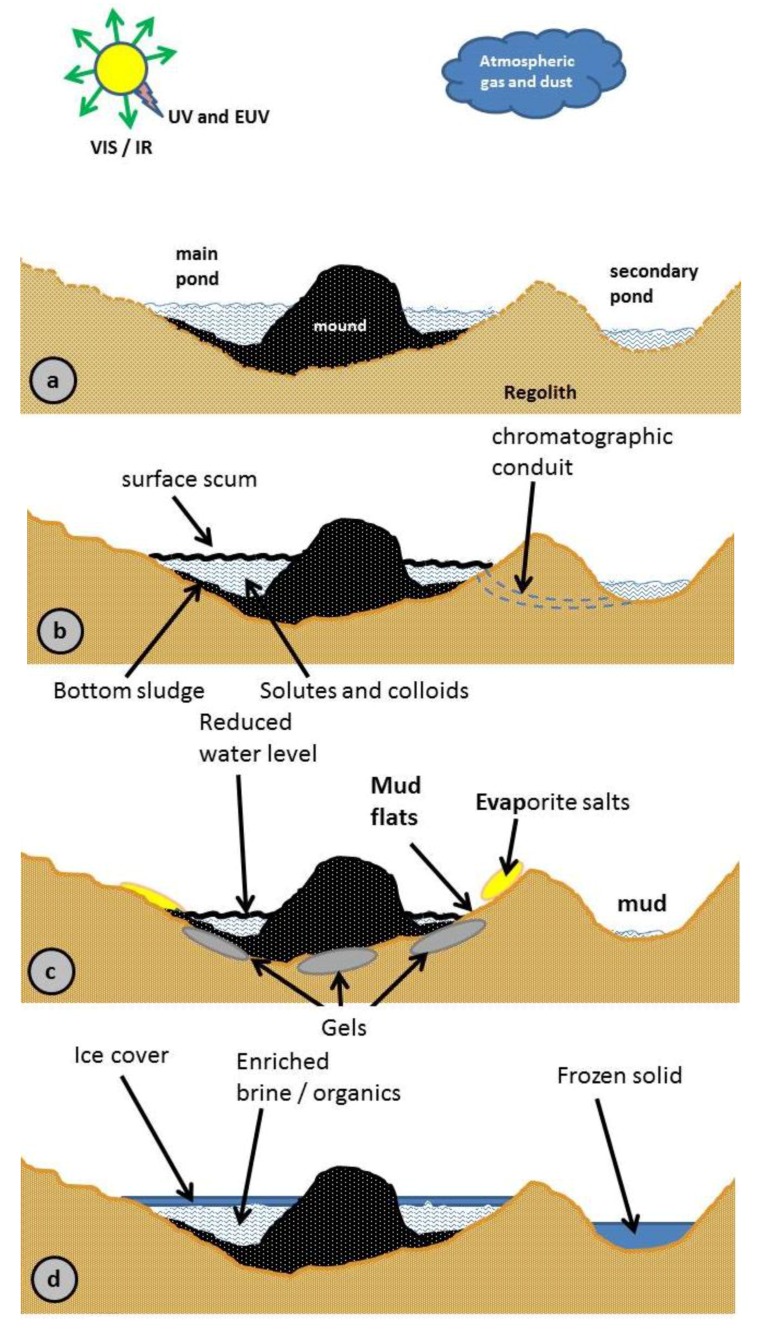

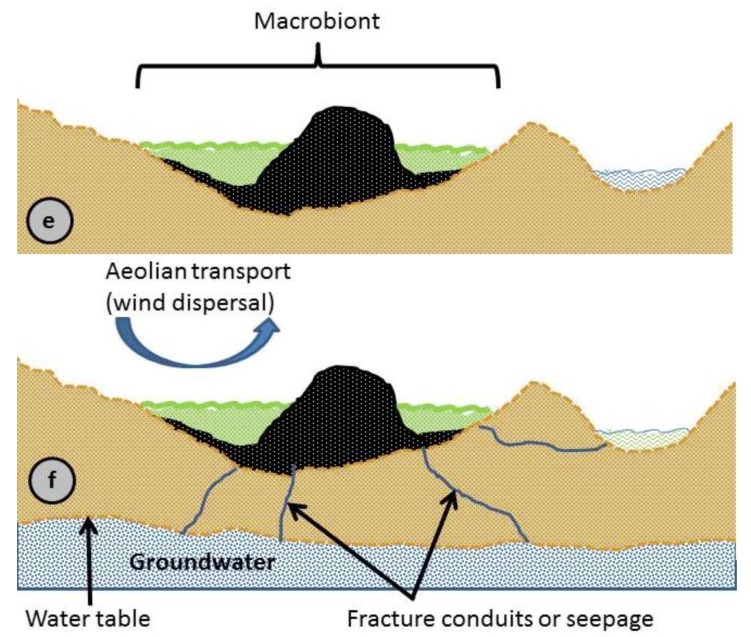
Comet pond phases (**a**) Schematic of early pond configuration, with satellite pond (s). (**b**) Gravitational force results in floating scum and sunken sludge (graded bed). (**c**) In a hot dry climate, evaporation will precipitate salts and form hydrogels. (**d**) In a cold climate, or at night, ice forms and freeze/thaw cycling can occur. (**e**) The Macrobiont forms (a consortium of living entities, see text). (**f**) Escape from the pond can occur via multiple paths, into the air or planetary hydrological network.

In Figure 3a, the previously published model is shown [1]. We now refer to this model as a generalized model.

Solar insolation provides warmth and photon energy, enabling primitive versions of photosynthesis as well as selective degradation of susceptible molecules. Surrounding regolith, saltating grains, and airfall dust provide planetary surface minerals, including soluble salts. Atmospheric gases, including photochemical products and volcanic emissions, can also react with pond constituents.

The added panels visually portray what was previously described only in words. In Figure 3b, a surface scum may form from organics with a density lower than the liquid medium (which may be salt-laden). EUV is attenuated by both surface scum and dissolved minerals (especially Fe) in the pond. Seepage through the mound and regolith provides natural chromatographic separations.

Evaporative losses affect the pond in multiple ways, concentrating some components and losing others, Figure 3c, while producing a graded shoreline with salts and mud flats. In addition to our previously discussed wet/dry cycles, a gel component is added [7,60,61]. The latter enables the preservation of chemicals during the dry cycle.

Further, we show the freeze/thaw model, Figure 3d. Such a cycle acknowledges the fact that comet landing could happen in various areas on the planetary target, including a high-latitude cold region. The freeze/thaw model is chemically important, since it enables preservation and aids the polymerization reactions of RNA [62,63,64]. In addition, the freeze/thaw model will select chemistry which is kinetically favored at low temperatures (low activation energy). This is in contrast to the reactions at high temperatures, which typically enable a large variety of chemical reactions to occur since sufficient energy is provided to overcome both higher and lower activation energies (e.g., [65]). A multitude of reactions typically lead to complex mixtures which often result in tar or asphalt-like phases. Such phases are not conducive to the chemical evolution of basic biochemical entities [66,67].

In Figure 3e, we show life evolving for the comet pond, which now serves as a Macrobiont [68,69]. A composite picture of the comet pond shown in these various figures leads to the important conclusion that life as it evolves in the Macrobiont can survive better against harsh environmental changes as it resides in the gel phase, or as it moves downwards or sideways into the ground waterways.

Figure 3f depicts dispersal modes in the comet pond, such as by wind, ground water, and overflow. The action of wind can levitate and widely disperse material. Wind can also drive waves to breach the pond boundaries or create new channels for stream erosion. Fractures or regolith porosity can allow the migration of organisms into the water table. All paths have the potential to eventually reach lakes or oceans.

#### 2.4.2. Geophysical and Physicochemical Environment

Temperature in the pond can fluctuate significantly during the natural diurnal cycle because the pond is small. Temperature extremes may be somewhat moderated because of the shorter day (~6 h for earliest times). There may also be different zones because of shallow versus deeper locations. If, as portrayed, there is a central mound of low-albedo cometary material, it could be subject to significant daytime heating and may have thermal inertia sufficiently high that the thermal gradient between the interior of the mound and the surrounding moat switches direction between day and night.

For a comet pond located at high latitudes or being formed during local wintertime, some or all of the water may become re-frozen. This may have significant advantages because it can create multiple freeze/thaw cycles which have advantages in prebiotic syntheses, including polymerization reactions, as discussed above. The pond will not freeze homogeneously because of several effects, including thermal heating from warmer soil below, the high heat capacity of water, convection patterns, a lower thermal capacity in shallow areas, ice-capping because of the lower density of ice, and so forth. Some shallow areas will be subject to repetitive freeze/thaw cycling on a diurnal frequency.

Acidity and basicity of the pond can be variable, both in space and time. The mafic minerals in equilibrium with H_2_O will push the pH modestly in the alkaline direction, potentially reaching values of 9 to 11. However, there are other components which can drive the system toward the acidic side during weathering processes. These include the sulfides, if there is sufficient oxygen available to create sulfates.

Rather than the cometary component providing acidity, however, it is also possible that the global volcanic emissions of S and Cl-containing gases can result in very low pH during weathering processes, as appears to be the case on Mars where ancient sediments contain minerals such as jarosite, which only forms at very low pH [70]. As pointed out in Knoll et al. [71], many prebiotic reactions reported in the literature are based upon neutral or alkaline conditions, and may not proceed well at low pH.

Certain natural separation and purification processes will contribute to the progression of the sequence of events necessary for coupling products at the most fruitful locations or times. For example, the central mound and/or the graded bed at the bottom and/or shorelines can all serve as natural liquid chromatographs as mixtures percolate through them.

Evaporites generally occur as spatially-ordered sequences as various specific salts precipitate out at different concentration levels. Reworking of evaporite beds by fresh infusions of water or brine can create multiple wet/dry cycling and additional heterogeneities. Geochemical redox or pH gradients in soils can produce strong separations from fluid loads.

Crystallization phenomena, or inhibition thereof, can result in the striking separation of phases. Preferential adsorption properties of clays and other minerals can sequester important ions and certain organic molecules.

In summary, because the pond is a partially open system interacting with a complex natural environment, it is subject to many of the processes that produce such variety and heterogeneity in purely geochemical systems.

## 3. Emergence of Life in the Pond

The pond itself is viewed as the environment having conditions favoring multiple prebiotic chemical reactions and diversification which can enable the rise of living systems. There are many aspects to this problem. The centerpiece is RNA, which is the essential ingredient of the RNA World hypothesis [72,73,74,75]. This theory postulates that RNA is the original self-replicator which had the dual capability to both carry the genetic information and to act as a catalyst in replication and energy utilization. Research by the Joyce group [76], Szostak group [77], and the laboratories of other groups, have actively pursued the goal of understanding and demonstrating how an early ribozyme can create an incipient RNA World that efficiently evolves to full-fledged life forms. However, our emphasis here is on the preceding conditions that make the rise of the proto-ribozyme molecules and other molecular systems necessary to support it possible. 

### 3.1. Relevance to Other Theories of Prebiotic Synthesis Leading to Life

We have reviewed numerous organic molecules and inorganic catalysts which are in comets and would form a rich and diverse pool of ingredients in the comet pond, highly suitable as starting materials for prebiotic chemistry. Additional organics could be available for the pond, notably those that are formed by atmospheric processes on the early Earth. What follows is a brief review of some important prebiotic chemical processes which are well suited for the comet pond milieu.

One example is the formation of amino acids, the building blocks of proteins, synthesized in the laboratory under reaction conditions that simulate lightning discharges in gases which were thought to be present in the atmosphere of the early Earth [3]. Miller’s experiment showed that organic compounds important for life can indeed be formed under prebiotic conditions. Many more experiments by different investigators have shown that starting from very simple molecules, such as those found in space (listed above), prebiotic synthesis of biologically relevant compounds can be accomplished [78].

Steady progress on prebiotic syntheses has been made since then. The subject is often reviewed [79,80]. Some examples are selected here.

Taking clues from present-day biology, de Duve proposed an early thioester world, in which thioesters had a role in primitive (proto) metabolism [81]. This proposal highlights the role of sulfur compounds in early prebiotic processes, which can be accommodated by the pond’s expected abundant inventory of sulfides and sulfonated organics.

In the “iron-sulfur world” theory of Wächtershäuser [82], prebiotic functions of carbon fixation from environmental gases proceed in intimate association with minerals, such as pyrite (FeS_2_). As reviewed above, the comet pond is well endowed with sources of Fe-S from the cometary material, even if the terrestrial site is lacking them.

Anoxygenic photosynthesis with Fe(II) as the electron donor using exclusively photosystem I has been proposed as being the earliest type of photosynthetic process [83]. Photoferroautotrophy would allow an anoxygenic iron-driven Fe(II) photosynthetic energy source for inorganic carbon (CO_2_) fixation early in the history of life [84]. The comet pond has abundant sources for Fe(II) from its sulfides, metals, and mafic minerals.

Various types of minerals can serve as substrates or outright catalysts in directing the path of organic reactions. With respect to prebiotic evolution, there has been notable work on montmorillonite smectite clays (common in deserts) by the Ferris group [85]. Other mineral systems, including double-layer hydroxides (relevant to the serpentinization process that also produces H_2_ from H_2_O), have been pursued by the Arrhenius group [86], and many other mineral systems in many other laboratories.

Prebiotic synthesis of biologically relevant compounds has continued to advance [87,88]. The prebiotic synthesis of RNA is extremely difficult, but much progress has been made.

Prebiotic analogs of RNA have also been considered. The molecular structure of RNA was varied with the objective to explain why RNA evolved chemically to its present structure. Benner synthesized sulfone-linked RNA [89], which is not charged, as opposed to the natural phosphate backbone. The sulfone-linked RNAs have been found, however, to be prebiotically unsuitable since they fold and aggregate in water. This shows the importance of charged backbones in nucleic acids, since charges prevent these undesired behaviors. Charged backbones are thus selected in nature. This also means that such charged structures need to be stabilized, in the simplest way by inorganic cations, which emphasizes the critical role of inorganics for the stabilization of RNA in the RNA world. The comet pond will be rich in cations from cometary material, but Ricardo et al. [90] and Benner et al. [66] have found that borate complexes are highly suited to the task of stabilizing the ribose component of RNA. The concentration of boron in carbonaceous chondrites and common terrestrial igneous rocks is very low, but in deserts, the processes of salt enrichment can facilitate the concentration of borax or other boron minerals. For example, in a residual lacustrine environment on Mars, boron enrichments of the order of thirty times have been found [91], which could of course be an indicator of much higher levels nearby.

The progress continues, with new innovative prebiotic syntheses of activated nucleotides by the Sutherland research group (e.g., [92,93,94,95,96]). These “cyanosulfidic” pathways are based on combining HCN with H_2_S, UV light, and the aid of a Cu catalyst. Not only can nucleotide precursors be synthesized, but precursors to about one-half the common amino acids and to lipids are also available via somewhat different conditions. As noted above, comets are endowed with HCN and other cyanogens, as well as Cu-bearing minerals. Copper enrichments also occur on Earth, and even with the limited exploration of Mars, so far, a sediment with more than a 20-fold enrichment of Cu has been discovered [97]. In additions to the HCN and H_2_S in comets, the planetary environment could provide H_2_S from its primitive atmosphere, as well as abundant UV light across a wide spectrum in the absence of oxygen and ozone. The comet pond is eminently well suited to this particular scenario.

As described above, in colder regions, the comet pond can undergo freeze/thaw cycles, which, based on recent findings, can promote the synthesis of RNA, in addition to preserving and stabilizing various molecules [62,63]. A further advantage for the comet pond, when in its freeze/thaw cycle, is that prebiotic chemistry in general in the water-ice matrix occurs readily [98]. The reason for this is that the organic materials and salts concentrate in the liquid phase of the crystalline ice-matrix and form eutectic solutions. For example, the methanol-to-water ratio in comets can be as high as 10% [32], and will be concentrated further as it serves as an anti-freeze to lower temperatures for the liquid not precipitated as H_2_O ice. Such concentration effects can increase reaction rates and specificity. 

Recently, progress has been made in prebiotic organic reactions in water as a reaction medium, and also in the solid state. The latter would be relevant for the desiccated pond, while the former would be applicable for the wet cycle of the pond. Most organic compounds are not water-soluble, but they still react in water, often quite rapidly. Recent findings show that such reactions occur “on water” [99]. This means that the reaction occurs when the insoluble organic materials are driven towards each other in water by their hydrophobic interactions. The proximity between molecules facilitates the reactions, and the selectivity and specificity of the reactions is often improved. Organic reactions in the solid state are also surprisingly successful and are often fast [99]. This all means that in the comet pond, active chemistry can occur both in the aqueous solution and in the solid state.

As discussed in the section on advantages of the comet pond milieu for prebiotic synthesis, great diversity in terms of pH’s, the presence of various clays which can act as catalysts, and purification of the reaction mixtures via circumstantial means (such as natural chromatography or phase-change separations) can be available. The comet pond may therefore be considered as an association of multiple, semi-isolated chemical reactors.

### 3.2. Concept of Macrobiont

The pond may also be viewed as a proto-organism, albeit one that is very large in size. In its course of progression, at some stage, it may achieve the capabilities within its boundaries of harnessing energy from the environment coupled with the synthesis of molecules of various complexities, including an encoded macromolecule which can serve as a genetic memory. In this way, it becomes a macrobiont.

Merging the two capabilities of information management and biased production of specific molecules may emerge slowly and inefficiently. Yet, once it is achieved, the pond may be considered as exhibiting the key properties of an organism. The pond cannot be considered an organism itself because there is no reproduction of the pond. But it would be the essential host environment for living entities.

At some point, earlier or perhaps late in the process, the ability for encapsulation would be invented. This would be favored in terms of the efficiency and the speed of reproduction because the necessary ingredients could be further concentrated and isolated from the dilutive or degradative actions of non-essential molecules. A natural advantage would accrue to the encapsulated entities, which would allow their numbers to increase at the expense of unprotected molecules.

Improvements in isolation and compartmentalization might evolve along the path of gels [100] or coacervates, eventually to micelles composed of amphiphilic molecules to form protocells [101]. It might not even be necessary that all protocells are equivalent or fully configured, as long as they are able to interchange products with complementary protocells. However, having distributed cooperative capabilities instead of consolidated ones will limit their ability to survive and prosper outside the confines of the pond unless they can form associations with one another. The stromatolite community of microorganisms may serve as a sophisticated example of this more primitive association by less capable entities. Other examples of intimate cooperation include multicellular organisms, as well as the eukaryotic cell containing isolated or sequestered functions in various organelles.

The pond may be viewed as an indispensable part of the earliest lifeform, providing the necessary environment for hosting various zones of partially compartmentalized functions. Catabolic and anabolic processes may be driven by the solar flux, by variations in thermodynamic equilibria in the ever-changing thermal regions, and/or by reactions with disequilibrium photochemical products from the atmosphere. 

The pond also exhibits other characteristics of a biotic entity. The pond itself is indeed bounded, as in a cell, but it is also distinctly non-locomotory. It also cannot reproduce. But, as the natural processes which originated in the segregated chemical pathways grind toward the inevitable conclusion of stagnation, the evolutionary pressure mounts for the natural selection of microminiaturization and juxtaposition of critical physiochemical processes. The macrobiont ultimately survives in the form of offspring of more efficient and prolific microforms.

The organisms created within the pond macrobiont may initially be larger than microscopic in size, except as limited by molecular forces, because they almost surely will host less efficient metabolic systems than modern life. However, their emigration from the pond into the planetary environment at large will be facilitated if they are smaller. For example, one method of dispersal is by wind, plucking organisms in aerosols and transporting them over comparatively large distances to other favorable environments where they may survive and reproduce.

Another pathway for emigration is through porosity in the regolith, along fractures in rock, or through fissures in the soil column down to the local water table. Once in the groundwater, they could be passively dispersed over long distances. Of course, survival underground requires special capabilities because they would no longer have access to solar illumination and would have restricted access to atmospheric gases. On the other hand, it is now well appreciated that there is an extensive underground biosphere which inhabits not just local groundwater, but also hydrologic systems at great depths in the Earth’s lithosphere [102]. In some cases, these communities exploit the geochemical serpentinization reaction which produces H_2_ or CH_4_ [103].

Yet another emigration pathway is the ephemeral or permanent streams and rivers which make up the surface hydrologic networks that eventually transport water and its particulate loads to the oceans. Once the pond overflows, due to recharge from rain or melting snow, or due to erosion of some area of its rim, some of its ingredients and/or their progeny can find their way to the far reaches of the planetary globe. Remaining at the surface enables a photoautotrophic existence and potentially even a solar-independent existence if atmospheric gases or complex organic molecules are available to enable chemolithoautotrophy or heterotrophy, respectively. Remaining in the euphotic zone within a deep ocean requires a degree of phototaxis, which requires both sensing and self-propulsion, or motility, which in turn invoke advanced degrees of molecular and physiological evolution. Inhabitation of tidal zones would of course be simpler, although the early Earth had far larger tidal excesses than today due to the ancient proximity of the Moon.

## 4. Advantages and Disadvantages of the Comet Pond Model

Compared to other models of the contribution of extraterrestrial organics to the origin of life, the comet pond hypothesis retains the high concentrations of a uniquely diverse set of organics that comprise the composition of pristine cometary material. This may be compared to dispersed quantities of carbonaceous chondrites, and especially to the ordinary chondrites, which may have supplied the final veneer of material to planet Earth [104]. On the other hand, some carbonaceous chondrites have sufficient strength to survive entry and reach the surface of Earth intact, without destroying their constituent organics from the heat of entry. This is the basis of the ponds and meteorites scenario of Pearce et al. [10], where it is hypothesized that with multiple entries and multiple natural ponds on Earth, these resulted in the creation of RNA molecules. However, the organics in asteroidal meteorites have been modified by various processes, including aqueous alteration on the parent body, and it has not been shown that immersion of such meteorites in water and exposing them to wet-dry cycles can produce the *de novo* synthesis of RNA.

Another recent hypothesis involving little ponds is the hydrothermal pools scenario of Damer [7]. In this hypothesis, the hot water pools in hydrothermal fields near volcanoes or other magma chambers are seeded by organics from natural terrestrial processes, as well as from in-fall of extraterrestrial organic-containing matter. The contributions from the former are unknown and perhaps unknowable and the contributions from the latter are subject to the double-dilution effect. Indeed, Pearce et al. [10] find that the contributions of organics from interplanetary dust particles are minor compared to those from carbonaceous meteorites. They also point out that the occurrence of hydrothermal pools is of the order of a hundred or thousand times less than the prevalence of natural ponds.

Other scenarios include the hypothesis that life began at a deep sea hydrothermal vent, partly because the most ancient organisms in the evolution of life include a preponderance of hyperthermophilic organisms [105]. This remains controversial and for a review of the case for a cold origin of RNA and hence life, consider for example, Feller [106]. On the other hand, there is nothing about the comet pond hypothesis that precludes a hot origin, except that either the whole Earth itself was hot when the pond was created, or the cometary object landed in a hydrothermal area. Even the latter does not guarantee a hot environment because soil insulation prevents high surface temperatures except where hot spring water or hot fumarolic gases are being released. The deep sea vent version is particularly complex because molecules formed in the vent will be swept into the surrounding ocean waters and suffer from the spatial dilution effects we and others consider so deleterious.

As alluded to above, the comet pond is also ideal for a cold origin of life, or a cold-to-warm origin, because it provides the starting material in frozen form (temperatures down to 160 K, or below). Depending on the details, the heat of entry may “soak back” to the landed material, or it may be mostly carried away by ablation. This scenario is therefore quite accommodating in terms of plausible thermal regimes.

Another advantage of the hypothesis is the melding of terrestrial constituents (as well as the physical environment) with the comet’s constituents. As discussed above, boron may be enriched and available in a desert landing location, but is at very low concentration in cometary matter. Clays and other useful minerals may also be supplied.

One disadvantage of this model is that it invokes a scenario which is of low probability, albeit of high potential importance. Establishing the order of magnitude of such a low probability is daunting. This will undoubtedly require extensive modeling of the phenomena of favorable entry/descent/landing scenario’s. Further advances in fidelity will also be needed in the Nice Model, Grand Tack model, and Late Heavy Bombardment duration and fluence to enable more accurate estimates of the integrated number of impacts of icy bodies from the deeper portions of the solar system onto the inner planets.

Another hindrance is that we do not yet have available bulk cometary material that includes intact organic components to assess the suitability of these materials as prebiotic precursors. This knowledge is presently incomplete and may be augmented by the further study of IDP’s and certain asteroidal bodies, but will not be complete until the return to Earth of a sample taken from the surface of a cometary nucleus.

## 5. Conclusions

A highly heterogeneous, shallow pond is hypothesized as a possible model for the origin of metabolic functions. The pond contains organic matter, some solubilized and some as condensed, absorbed, or occluded phases in particulates. The composition of the matter is highly diverse, both chemically and physically. Depending upon the particle size, density, and hydrophobic interactions, some particles float and form a surface scum. Others sink to form a graded bed, and still others are colloidal in size and remain suspended for long periods of time. Solar heating and advective cooling provide thermal gradients which drive convection currents to maintain the transport and suspension of the material, as well as some aggregations on the pond floor. Wind action causes the redistribution of matter, and provides a supply of exogenous mineral grains. Ultraviolet radiation-induced polymerization and degradation continuously processes near-surface organic material. Chemical weathering of minerals at the pond bottom, pond shorelines, and of eolian sedimented matter brings fresh ions into the solution and provides new catalytic mineral forms and particle surfaces.

It is supposed that in the course of this stochastic sequence of events, semi-isolated chemical regimes form. Dynamic feedback chemical processes, such as in a natural reflux reactor, cause enhanced conversion and synthesis of various compounds. Autocatalytic situations occur. For example, high molecular weight material selectively binds to stereo-specific sites on particulates situated on the pond bottom, thereby being withdrawn from the circulation. Other material, being unbound, is eventually exposed to the UV and gas environment of the near surface.

Meteorological long-term cycles and episodic events produce dehydration-rehydration and freeze/thaw chemical separations and physical migrations. The pond may be viewed as a macrobiont, with various semi-isolated zones as loosely compartmentalized pseudo-organelles. Like a cell, it exhibits energy capture, chemical conversion, and a boundary, but is non-locomotory and cannot reproduce itself. The macrobiont ultimately survives in an offspring of more efficient and prolific microforms, which disperse over the planetary body via eolian and hydrologic pathways.

Although the intact landing of pristine cometary material is a very low probability event, the preponderance of comets in our solar system, and the indications that other planetary systems also are rich in comets (exo-comets), argues that this possibility should be taken seriously. A recent objective study has documented the existence of a positive bias among the general public that the discovery of an extraterrestrial origin of life is an expected event, concluding that “perhaps such news causes people to take comfort in the fact that we are not alone in the universe” [107]. The same tendency is also anecdotally noted in the scientific community, although there is no objective evidence available at this time that life is abundant in the universe. As scientists, with our objective methodologies, we should remain open to the possibility that the formation of living entities is in fact an exceptionally rare occurrence, and very special circumstances may be necessary to create them.

Much has been learned about cometary matter in the time period since this model was first proposed [1], but much remains to be discovered. Upcoming missions to carbonaceous asteroids may shed new light on the varieties of organic matter in our solar system. The Hayabusa 2 mission (http://global.jaxa.jp/projects/sat/hayabusa2/) will return a bulk sample of the asteroid Ryugu in 2020, and the OSIRIS-REx mission is scheduled to return a bulk sample from the asteroid Bennu in September of 2023 [108]. Of three comet samples return missions recently proposed to NASA (CAESAR, CONDOR, and CORSAIR) for its New Frontiers IV mission, the CAESAR mission has been selected for detailed preliminary study. Its mission timeline calls for the return of a bulk sample from the surface of the comet 67P/Churyumov–Gerasimenko in 2038.
